# Towards Independent Control of Multiple Magnetic Mobile Microrobots†

**DOI:** 10.3390/mi7010003

**Published:** 2015-12-28

**Authors:** Sagar Chowdhury, Wuming Jing, David J. Cappelleri

**Affiliations:** Multiscale Robotics and Automation Laboratory, School of Mechanical Engineering, Purdue University, West Lafayette, IN 47907, USA; sagar353@purdue.edu (S.C.); jing6@purdue.edu (W.J.)

**Keywords:** magnetic microrobots, path planning, mobile microrobots

## Abstract

In this paper, we have developed an approach for independent autonomous navigation of multiple microrobots under the influence of magnetic fields and validated it experimentally. We first developed a heuristics based planning algorithm for generating collision-free trajectories for the microrobots that are suitable to be executed by an available magnetic field. Second, we have modeled the dynamics of the microrobots to develop a controller for determining the forces that need to be generated for the navigation of the robots along the trajectories at a suitable control frequency. Next, an optimization routine is developed to determine the input currents to the electromagnetic coils that can generate the required forces for the navigation of the robots at the controller frequency. We then validated our approach by simulating an electromagnetic system that contains an array of sixty-four magnetic microcoils designed for generating local magnetic fields suitable for simultaneous independent actuation of multiple microrobots. Finally, we prototyped an mm-scale version of the system and present experimental results showing the validity of our approach.

## 1. Introduction

Manipulation of micro- and nanoscale objects is considered the enabling step for many biological and manufacturing tasks that might potentially revolutionize the respective industry. For example, manipulation of cells to form a pattern can enable cell based assembly, study of cell behavior in a group, diagnosis for therapy, *etc*. On the other hand, the ability to assemble heterogenous microscale components into an intricate functional device can potentially benefit energy, communication, and computing industry.

Microfluidics [1], electrostatic [2], magnetic manipulation [3,4], Atomic Force Microscopy (AFM) [5], optical tweezers (OT) [6,7,8], fixed manipulators [9,10], and micro-grippers [11] are some of the enabling technologies proposed for micro and nanoscale manipulation. Many of these technologies (e.g., OT, magnetic manipulation, *etc*.) use small agents that can be treated as robots to manipulate microscale objects. The effectiveness of these systems rely on their ability to control multiple agents (robots) independently to accomplish multiple tasks in parallel. The following are some of the approaches researchers have developed to control multiple robots independently.

Diller *et al.* [12] developed several reconfigurable magnetic micromodules (Mag-*μ*Mods) for assembly and disassembly operations. They achieved independent locomotion of these modules by using electrostatic surfaces. By selectively activating a particular surface, the attached robot-module can be immobilized while the other modules can be moved using the gross magnetic field generated by six coils. Each module is a permanent magnet which limits its application only to the assembly of the objects that are magnetic in nature. In another work [13], they demonstrated the pushing based manipulation with Mag-*μ*Mods. However, the magnetic field generated by six coils cannot be controlled locally. Instead, multiple heterogeneous Mag-*μ*Mods are manufactured that respond differently to the same magnetic field. By utilizing their dynamical behavior in response to the same magnetic field, the robots can be controlled independently. Since the authors have relied on global magnetic field to actuate the robots, the control is somewhat coupled and cannot be scaled for large number of robots.

Pelrine *et al.* [14] developed a swarm of robots arranged on a printed circuit board (PCB). Each robot is a mm-scale magnet and is actuated by magnetic field generated by a PCB. The PCB generates localized magnetic fields for individual control of the robots. Using the swarm of robots, massive parallelization in assembly operation can be achieved. The system is capable of fast manipulation. The demonstration showed 73 robots performing coordinated moves each at 19 moves/s. The total system rate is 1386 (∼ 73 × 19) moves/s.

Optical tweezers (OT) have been utilized both for microscale assembly [15,16] and biological manipulation [17,18]. Highly focused laser beams are used to move and orient parts in 3D with high precision. By coordinating multiple laser beams highly precise assembly tasks can be realized. Chowdhury *et al.* [19] used yeast cells as microrobots by actuating them using optical tweezers. They have developed graph search based algorithms for automated manipulation of multiple yeast cells independently towards their respective goal locations in the presence of external fluid force field inside a microfluidic chamber. The yeast microrobots are capable of avoiding obstacles randomly moving in the workspace during the course of navigation. A number of planning and control approaches to realize automated cell manipulation using optical tweezers have also been developed by [20,21,22]. Hu *et al.* [23] utilized the heating energy of laser to control the movements of bubbles for manipulation and assembly of microscale objects. However, OT based assembly operation suffers from slow speed. Moreover, the small workspace (100 µm × 100 µm) makes it more suitable for biological manipulation than manufacturing applications.

Magnetic manipulation [24,25,26] is regarded as a promising technology due to its ability to generate a force ranging from pN to *μ*N, cheap installation, and precision in operation. However, most of the magnetic manipulation setups are designed to create a global magnetic field that can severely affect the flexibility of operation [27,28,29]. The ability to automatically control multiple magnets independently can enable high throughput operation.

**Figure 1:**
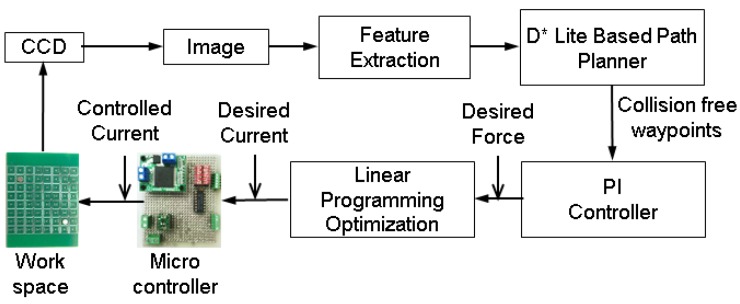
The overall approach: The approach consists of tight integration of perception, planning, control, and optimization.

In this paper, we have developed a specialized substrate that is suitable to be scaled down to generate local magnetic field to control multiple microrobots independently. Moreover, we have developed an approach for automatic navigation of single and multiple magnetic robots in an environment with moving obstacles. Our approach consists of three steps (Figure 1): (1) computing collision-free trajectories for the robots; (2) determining the required forces to move the robots with the help of controllers; and (3) optimizing the input currents to the coils to generate the required forces. We have also proposed a new design of an array of planar microcoils that can actuate multiple robots independently to demonstrate our approach.

## 2. System Architecture

We have demonstrated our planning approach on a setup which is capable of actuating multiple robots independently with magnetic forces. Most of the magnetic field based actuation systems are global in nature and hence can control only a single robot independently [24,25,26].

To overcome the limitation of global magnetic field in controlling multiple robots independently [30], we have developed a specialized substrate with an array of planar microcoils. Each planar microcoil has a winding width of 178 µm, an out-of-plane winding thickness of 35 µm, and a winding spacing of 178 µm with five turns. Each winding is rectangular in shape for the ease of fabrication. For simulation, we have approximated them with concentric rectangular coils with equivalent lengths. Each coil is capable of generating a local magnetic field that is dominant only along the area of the coil. We have used standard PCB (printed circuit board) technology to fabricate the array of microcoils. Each coil is connected to a power source at the bottom through a via. The current in each coil can be controlled independently in both magnitude and direction through individual connections with custom control electronics.

**Figure 2:**
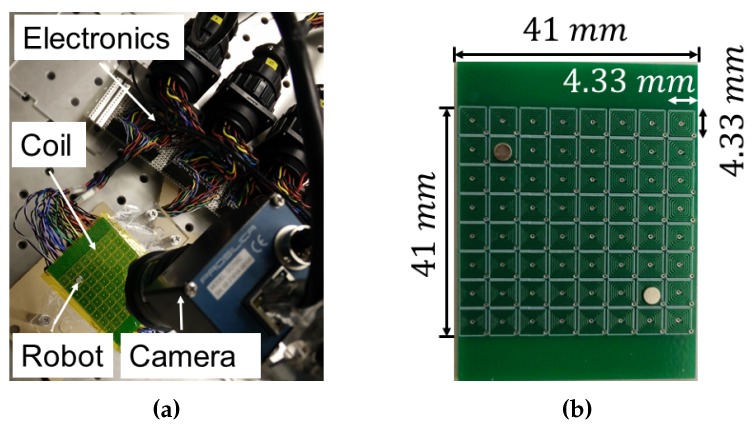
Experimental setup: (**a**) overall setup with integrated components, (**b**) closeup of the microcoil substrate.

Figure 2a shows the experimental setup. An overhead camera (Prosilica, Allied Vision) is used to give the visual feedback of the state of the robot at a particular time instant in the workspace. The current in each coil can be controlled through a custom control unit. Each coil is designed to carry a maximum current of one amp. However, only the coils in the vicinity of the robot remain in action at a certain point of time. All of the 64 coils will not operate simultaneously, which would require a maximum current supply of 64 amps. The current in each coil is controlled by controlling the polarity and the voltage amount with a pulse width modulation (PWM) generator. An Arduino microcontroller is used to send the PWM signal to the generator. The Arduino is connected to a CPU with an Intel^®^ Core${}^{\text{TM}}$ i7-4771 (3.50 GHz) processor and 16 GB RAM. All the computation is performed on the CPU and the calculated PWM duty cycles are sent to the Arduino. The Arduino sends the signal to the PWM generator which controls the current to the coils. Figure 2b shows a close-up of the specialized substrate with the microcoil array. Each coil can induce an attractive or repulsive force on the robot depending on the polarity of the PWM signal. The amount of force depends on the strength of PWM signal as well as the magnetic susceptibility of the robot material. We have used a permanent magnetic disc of diameter 2 mm and thickness 750 µm made of Neodymium as a robot.

## 3. Path Planning

### 3.1. Problem Formulation

**Given:**

- Initial states ${\left\{x_{i,\mathrm{init}}={\left[x_{i},y_{i}\right]}^{T}\right\}}_{i=1}^{n}$ of *n* robots to be transported in *X*, where *X* is the discretized operating space of the entire magnetic operating field;
- Goal states of *n* robots ${\left\{x_{j,\mathrm{goal}}\right\}}_{j=1}^{n}$ represented as grid locations within *X*;
- Static and dynamic obstacles ${\left\{\Omega_{k}\right\}}_{k=1}^{l}$ represented either as other objects or other moving robots.

**Find:**

- Collision-free paths ${\left\{\tau_{i}\right\}}_{i=1}^{n}$ for *n* robots to move their goal states ${\left\{x_{j,\mathrm{goal}}\right\}}_{j=1}^{m}$.

### 3.2. Approach

Path planning approaches for robot can be divided into two broad classes [31]: (1) Planning with perfect sensor information and (2) planning in uncertain environment. One popular approach is to discretize the workspace into configuration space with the application of graph search [32]. However, this approach gets computationally expensive for high degree of freedom (DOF) robots. Sampling based search algorithms (rapidly-exploring random tree (RRT), probabilistic roadmap motion planning (PRM)) [33,34,35] greatly reduce the computational burden. However, our planning problem is two dimensional and hence we use a graph search based algorithm in this paper.

We use a heuristic graph search algorithm D* Lite [36] for our path planner that can efficiently compute a collision free path for the *i*-th robot from initial state $x_{i,\mathrm{init}}$ to the respective goal state $x_{i,\mathrm{goal}}$. The algorithm is very fast for the 2D workspace that we are dealing with in this paper and functions like a backward version of the A* algorithm [32] where the states are incrementally expanded from $x_{i,\mathrm{goal}}$ to $x_{i,\mathrm{init}}$. The other robots and the objects in the scene are regarded as obstacles for the search. We have developed a heuristic to guide the search that can compute the collision free path with expansion of minimum number of states. Rather than starting the search from scratch every time the environment changes, the planner maintains an open set *O* which contains the states that are more likely to be expanded in the following steps, ranked by their costs. The planner utilizes the open set for replanning and focuses on the states that have a change in costs throughout the entire planning horizon. The planner inserts the states with change in costs due to the change in operating space *X* into *O* and continues expanding the states based on the lowest costs until a new path is determined. This provides efficient replanning for multiple robots navigating in a dynamic environment.

### 3.3. State-Action Space Presentation

The state space of the magnetic workspace is represented as a 2D rectangular grid since we move robots only in the *x*–*y* plane. The discrete state $x^{k}=\left[x^{k},y^{k}\right]$ of a robot is thus defined as a vector of its position at the time step *k* that corresponds to a particular grid cell.

**Figure 3:**
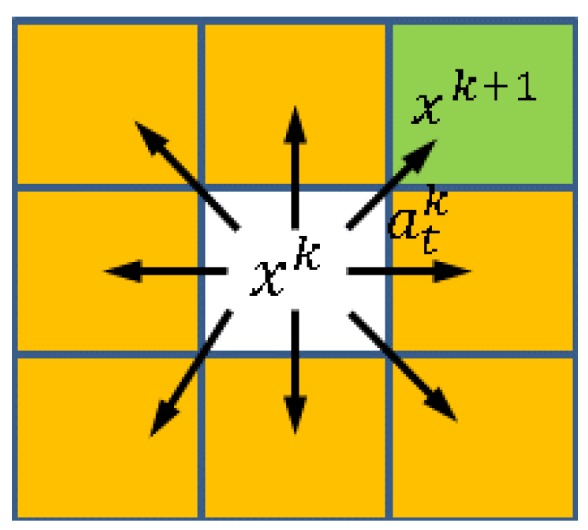
State-action space representation: The action set *A* consists of eight linear action $a_{t,i}^{k}$.

An action set $A=\{a_{t,1}^{k},a_{t,2}^{k},\ldots ,a_{t,8}^{k}\}$ consists of eight linear translation actions $a_{t,i}^{k}$ available for execution at a given time step *k* (Figure 3). All linear actions can be represented as follows:

(1) $$a_{t}^{k}\left(\delta x^{k},\delta y^{k}\right)=\left[\begin{array}{c} \delta x^{k}\\ \delta y^{k} \end{array}\right]$$

where δx and δy are the linear translations along *x* and *y* axis, respectively.

When the magnetic field executes an action $a_{t}^{k}$ at time step *k*, it transitions from $x^{k}$ to $x^{k+1}$ (Figure 3) using the following equation:

(2) $$x^{k+1}=x^{k}+a_{t}^{k}$$

### 3.4. Cost Function

The states from the priority queue *O* are expanded incrementally with their key values [36] computed as follows:

(3) $$\begin{array}{ccc} kv(x) & = & \left[kv_{1}\left(x\right),kv_{2}\left(x\right)\right]\\ & = & [min\left(g\left(x\right),rhs\left(x\right)\right)+h\left(x_{\mathrm{init}},x\right)\\ & & min(g(x),rhs(x))] \end{array}$$

where g(x) is the optimal cost-to-go from x to $x_{\mathrm{goal}}$, $h(x_{\mathrm{init}},x)$ is the heuristic cost estimate of the path between x and $x_{\mathrm{init}}$, and rhs(x) is the one step look-ahead cost which is calculated as follows:

(4) $$rhs\left(x\right)=\left\{\begin{array}{cc} 0, & \mathrm{if}x=x_{\mathrm{goal}}\\ min_{x^{\prime }\in succ\left(x\right)}\left(t\left(xx^{\prime }\right)+g\left(x^{\prime }\right)\right) & \mathrm{otherwise} \end{array}\right.$$

where succ(x) denotes a set of possible resulting states $x^{\prime }$ after taking an action a at state x and $t(x,x^{\prime })$ denotes the transition cost between x and $x^{\prime }$. In order to ensure optimality, the heuristic function should not overestimate the true cost to $x_{\mathrm{init}}$. The heuristic $h(x_{\mathrm{init}},x)$ computes the traveled distance for the robot to move between x and $x_{\mathrm{init}}$. We have used the Eucledian distance between x and $x_{\mathrm{init}}$ as a measure of $h(x_{\mathrm{init}},x)$.

We have also utilized the Euclidean distance between x and $x^{\prime }$ to calculate the transition cost $t(x,x^{\prime })$ since we are interested in computing the collision-free shortest path. Hence, $t(x,x^{\prime })$ is formulated as follows:

(5) $$t\left(x,x^{\prime }\right)=d\left(x,x^{\prime }\right)$$

where $d(x,x^{\prime })$ is the Euclidean distance between x and $x^{\prime }$.

## 4. Controller Design

### 4.1. Problem Formulation

In this section, we describe a controller to control the applied magnetic force that is required for the robot to track a waypoint $w_{p}$ computed in Section 3. The torque to control the orientation of the robot can be derived similarly and is a focus of our future work. Here, we assume circular robot geometries with no preferred orientation. The control problem can be derived as follows:

**Given:**

- The dynamics $m\ddot{x}+\gamma \dot{x}+F_{fric}=F_{mag}$ of the *i*-th robot, where *m* is the mass of the robot, *γ* is the drag force coefficient of the surrounding medium, $F_{mag}$ is the driving magnetic force, $F_{fric}$ is the surface frictional force, and i=1,2,3,⋯n;
- A reference state $x_{r}\in {\left\{\tau_{i}\right\}}_{i=1}^{n}$ the robot needs to follow;
- A measurement $x_{m}$ of the robot state.

**Find:**

- A feedback control input $F_{f}$ to determine the required magnetic force such that the robot can follow the reference state $x_{r}$.

### 4.2. Approach

The goal of the controller is not necessarily to move the robot from one coil to another but to move the robot in any arbitrary direction in the workspace. With the controller, we can not only control the position of the robot but we can also control its velocity and acceleration. Hence, we can eliminate the overshoot of the robot unlike in the case of a simple on/off controller.

The components of the resultant magnetic force $F_{mag}$ on the robot from the set of the coils can be written as $F_{x}$, $F_{y}$, and $F_{z}$. It is assumed that the robot will be operating in a liquid environment. The dynamic behavior of the robot is influenced by several external forces, e.g., van der Waals, electrostatic, frictional forces, *etc*. The van der Waals and electrostatic forces can be determined with proper experimental procedure. However, we only consider frictional forces for the modeling in this paper since that can be determined from the surface material properties. The frictional force which acts against the direction of motion changes from rest to motion. The robot needs to overcome static friction when starting from rest. On the other hand, dynamic friction comes into play when the robot is in motion. Both the frictional forces can be expressed as follows:

(6) $$F_{fric}=\left\{\begin{array}{cc} \mu_{s}\left(W-F_{z}\right) & \text{static}\;\text{friction}\\ \mu_{k}\left(W-F_{z}\right) & \text{dynamic}\;\text{friction} \end{array}\right.$$

where *W* is the weight of the robot, $\mu_{s}$ and $\mu_{k}$ are the static and dynamic friction coefficients respectively, which depend on the contact surfaces between robot and the workspace. We have considered the contact between the robot and the surface as metal-metal contact and used the coefficients described in [37]. To track a reference state $x_{r}$, we apply a PI controller (proportional plus integral) to derive the required magnetic force as follows:

(7) $$F_{f}=k_{p}\left(x_{r}-x_{m}\right)+k_{i}\int_{0}^{t_{c}}\left(x_{r}-x_{m}\right)dt_{c}$$

where $x_{m}$ is the measured current position of the robot, $t_{c}$ is the control frequency, $k_{p}$ is the proportional gain and $k_{i}$ is the integral gain, respectively.

## 5. Computation of Currents

### 5.1. Overview

The robots used for navigation are magnetized objects. In this paper, we only focused on controlling the position of the robot. Hence, we just have to control the magnetic force $F_{mag}$. However, the orientation can be controlled by regulating the magnetic torque in a similar fashion. When placed in a magnetic field, the robots experience a magnetic force which is the driving force for moving a robot in a specified trajectory. The interaction between the robot and the magnetic field can be described as follows:

(8) $$F_{mag}=V_{r}\left(M\cdot \nabla \right)B\left(x,y,z\right)$$

where $V_{r}$ is the volume of the robot, M is the magnetization of the robot, B is the magnetic flux produced by the coils, and $F_{mag}$ is the force experienced by the robot. M is related to the magnetic flux B through the following relationship:

(9) $$M=\frac{\chi B}{\mu_{0}}$$

where *χ* is the magnetic susceptibility of the robot material and $\mu_{0}$ is the permeability of the free space (4*π*×$10^{-7}$ Hm${}^{-1}$).

**Figure 4:**
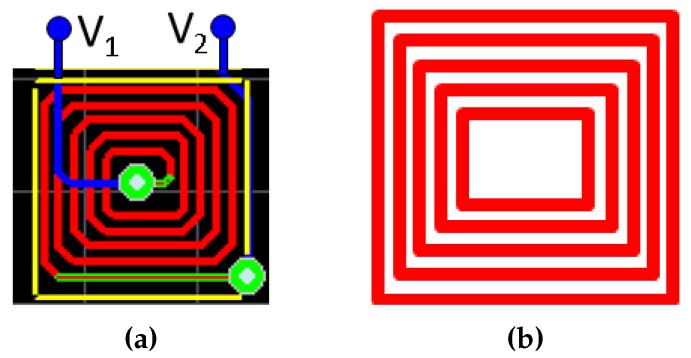
Rectangular approximation of the coil for simulation: (**a**) full view of the design of the coil; (**b**) concentric rectangular approximation of the coil.

**Figure 5:**
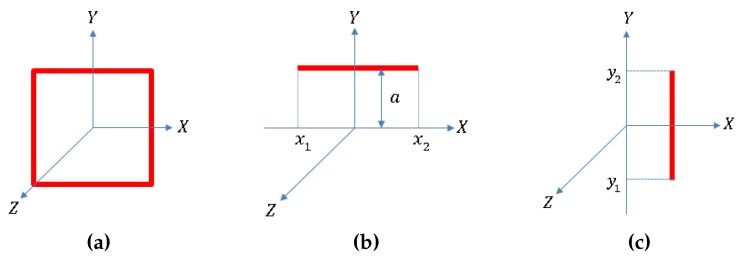
Magnetic flux calculation of a rectangular section of the coil: (**a**) a rectangular section of the coil with the coordinate axes; (**b**) horizontal section of the coil; (**c**) vertical section of the coil.

The planar microcoils are approximated as concentric rectangles with varying lengths for the simulations (Figure 4). The magnetic flux $B=[B_{x},B_{y},B_{z}]$ due to a horizontal segment (Figure 5) of the rectangular coil at a location P(x,y,z) can be calculated as follows:

(10) $$B_{x}=0$$

(11) $$B_{y}=-\frac{\mu_{0}zI_{c}}{4\pi \left[{\left(a-y\right)}^{2}+z^{2}\right]}\times \left[\frac{x_{2}-x}{\sqrt{{\left(x_{2}-x\right)}^{2}+{\left(a-y\right)}^{2}+z^{2}}}-\frac{x_{1}-x}{\sqrt{{\left(x_{1}-x\right)}^{2}+{\left(a-y\right)}^{2}+z^{2}}}\right]$$

(12) $$B_{z}=-\frac{\mu_{0}\left(a-y\right)I_{c}}{4\pi \left[{\left(a-y\right)}^{2}+z^{2}\right]}\times \left[\frac{x_{2}-x}{\sqrt{{\left(x_{2}-x\right)}^{2}+{\left(a-y\right)}^{2}+z^{2}}}-\frac{x_{1}-x}{\sqrt{{\left(x_{1}-x\right)}^{2}+{\left(a-y\right)}^{2}+z^{2}}}\right]$$

where $I_{c}$ is the current passing through the coil segment and the parameters $x_{1}$, $x_{2}$, and *a* are as defined in Figure 5. The magnetic flux in vertical segment can be calculated similarly [38]. With the vector summation of the magnetic flux for all the segments of the concentric rectangular sections, we can calculate the resultant flux due to a planar coil.

The goal of this section is to develop an approach to compute the required currents $I={\left\{I_{l}\right\}}_{l=1}^{m}$ in *m* coils in the vicinity of the robot that can drive the robot along the specified direction. The Equation (8) to Equation (12) suggest that there is a non-unique solution to this. Thus, we have formulated an optimization problem to determine the best solution.

### 5.2. Optimization Problem Formulation

The goal is to minimize the total amount of current in the coils that can generate the required force $F_{f}$ computed by the feedback controller in Section 4.1. The coils cannot withstand high currents since high currents generate heat which might burn the coils. Hence, our goal is to minimize the current that is needed to generate enough force to drive the robot. Moreover, the rapid fluctuation in current affects the reliability of the coils. Minimizing the total current ensures uniform distribution of the current. The overall optimization problem can be summarized as follows:

(13) $$\begin{array}{cccc} & \underset{I}{\text{minimize}} & & f_{0}\left(I\right)=\sum_{l=1}^{m}I_{l}\\ & \text{subject}\;\text{to} & & F_{mag}\geq F_{f}\\ & & & I_{min}\leq I_{l}\leq I_{max},\;l=1,\ldots ,m \end{array}$$

### 5.3. Approach

We have cast the optimization problem in Equation (13) as a linear programming problem. The constraint is applied as an inequality constraint since Equation (8) is a function of I. We have used the Matlab optimization toolbox to solve for optimized currents in real time.

## 6. Results and Discussion

### 6.1. Simulation Results

We have conducted extensive simulation experiments to demonstrate the effectiveness of our approach. Two representative simulations are described in this section. The planning frequency is set at 50 Hz whereas the controller and optimization loop are run at a frequency of 100 Hz. Since every instance of the planning algorithm is launched separately for the respective robot, the planning and control frequencies are scalable for a higher number of robots as long as there is sufficient computational power available. Moreover, the power of parallelization can be utilized since every instance of the planning algorithm is independent of each other. The noise is modeled by drawing a number from a Guassian Distribution with mean 0 and standard deviation 20 µm. The planner gives the next waypoint and the controller computes the required force to track the waypoint. Finally, the optimization loop computes the currents in the coils to generate the force. The parameters used for the simulations are shown in Table 1. The parameters are determined by experimental characterization of the system [28]. The drag coefficient is calculated using the following equation [39]

(14) $$\gamma =\frac{1.328}{\sqrt{Re}}$$

where, Re is the Reynold’s number which can be computed as $Re=\frac{v_{r}L}{\nu }$. $v_{r}$ is the velocity of the robot at a particular time instant, *ν* is the kinematic viscosity of the surrounding fluid, and *L* is the characteristic length of the robot. We have used mineral oil with $\nu =1.8\times 10^{-5}$ m${}^{2}$/s as the working fluid since it gets vaporized easily and hence is less prone to contamination. In order to replicate the real world scenario, we have introduced Gaussian noise into the measured states of the robot to test the effectiveness of the controller.

**Table 1:** Simulation Parameters.

Parameters	64-Coil System
Static Friction Coefficient, $\mu_{s}$	0.30
Dynamic Friction Coefficient, $\mu_{k}$	0.15
Number of turns, $n_{c}$	10
Permeability of air, $\mu_{0}$ (N/m${}^{2}$)	$1.26\times 10^{-6}$
Current limit (A), $I_{min}$ –$I_{max}$	0-5
Susceptibility of robot, *χ*	0.05
Robot diameter (mm)	2.0

**Figure 6:**
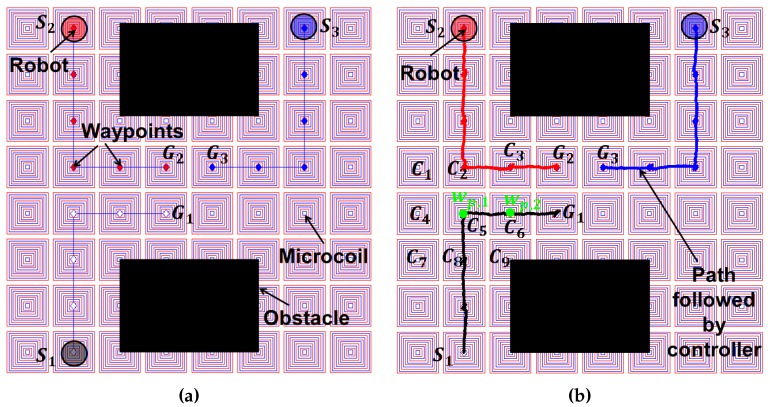
Autonomous navigation of three robots under the influence of local magnetic fields generated with the array of 64 microcoils: (**a**) collision free waypoints computed by planner (the action set consists of four linear actions (top, bottom, right, and left)); (**b**) paths followed by the controller with the presence of measurement noise.

In the first experiment, three robots are navigated autonomously on a workspace consisting of obstacles. To move multiple robots independently, an array of microcoils have been modeled (Figure 6). The coils are designed as rectangular shapes for the ease of fabrication. During the computation of the collision-free paths for a robot, the surrounding robots as well as the static objects are considered as obstacles. Since the surrounding robots (obstacles) are always moving, the environment for the planning is dynamic in nature. Hence, the planner utilizes the re-planning advantage of the D* Lite algorithm (Section 3) to compute the path efficiently. We have considered four actions (top, bottom, right, and left) that can take the robot to a neighboring grid in a discretized workspace. The adjacent four magnetic coils are taken into consideration during the optimization step to compute the required currents since they have the maximum influence on the robot. The magnetic field created by the distant magnetic coils are negligible and hence are turned off for the particular action. The initial and goal locations are marked as $S_{i}$ and $G_{i}$ respectively where i=1,2,3 (Figure 6a). The collision free waypoints are marked by “♢”. The respective controller for each robot tries to track the waypoint at the controller frequency which suffers from a measurement noise. The paths followed by the controller are shown in Figure 6b.

Table 2 shows the simulation results in one control loop for navigating between two waypoints $w_{p,1}$ and $w_{p,2}$. Both the waypoints $w_{p,1}$ and $w_{p,2}$ are located horizontally and the robot is at $w_{p,1}$ (Figure 6b). The coil system can generate a large force with a small amount of current since the robot is located much closer to the coil. Larger robot volume corresponds to larger force too. However, larger volume will have a larger static friction to overcome. All of the nine coils adjacent to the robot denoted by $C_{i}$ (i=1,2,3…9) in Figure 6b are activated to navigate the robot to the waypoint $w_{p,2}$ from $w_{p,1}$. The required forces and currents in the subsequent coils computed from the model described in previous sections are listed in Table 2. The negative currents generate repulsive force whereas positive currents try to pull the robot.

**Table 2:** Optimization Parameters.

$F_{x}$ (μN)	$F_{y}$ (μN)	$I_{1}$ (*A*)	$I_{2}$ (*A*)	$I_{3}$ (*A*)	$I_{4}$ (*A*)	$I_{5}$ (*A*)	$I_{6}$ (*A*)	$I_{7}$ (*A*)	$I_{8}$ (*A*)	$I_{9}$ (*A*)
1.2	0	−0.4	−0.6	0.4	−0.5	−0.8	0.8	−0.5	−0.6	0.4

### 6.2. Experimental Results

We have demonstrated the automated path planning approach as well as independent control of multiple robots with two physical experiments. The action set of the robot is restricted to four directions (left, right, top, bottom).

**Figure 7:**
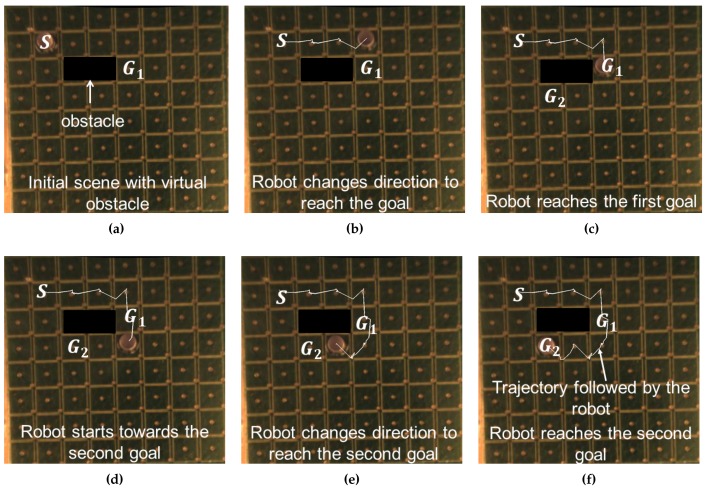
Autonomous navigation of a robot towards two goal locations on an array of 64 microcoils: (**a**) initial scene with the robot and goal location; (**b**) robot reaches a waypoint on its way to the first goal location $G_{1}$; (**c**) robot changes direction to reach the first goal location; (**d**) robot starts moving to the second goal location $G_{2}$; (**e**) robot changes direction towards the second goal location $G_{2}$; (**f**) robot reaches the second goal location $G_{2}$. The trajectory followed by the robot is marked with white line.

In the first experiment, we have demonstrated the automated navigation of a single robot towards multiple goal locations with the presence of virtual obstacles. The goal locations are dynamically assigned to the robot and the robot computes the path waypoints for navigation on real time. Figure 7 shows the automated navigation of a robot towards goal locations. At this point, we select the goals such that the robots cannot come very close to each other. Ideally, we do not need to make the robot from permanent magnets. We can make them out of ferro-magnetic materials. In that case, we can bring the robots much closer to each other. The robot starts from the initial location “*S*”. Due to the obstacle, the robot chooses to go to the right to reach the first goal location. The overhead camera sends the current location of the robot. Based on the robot location and desired waypoint, the currents in all the surrounding nine coils of the robot are calculated. The robot changes the direction to reach the first goal location “$G_{1}$”. The goal location is then changed to a second one (“$G_{2}$”) dynamically after the robot reaches the first goal (“$G_{1}$”). The path is recomputed to reach the second goal location “$G_{2}$”. Finally, the robot stops after reaching the goal location “$G_{2}$”. The white lines in the figure indicates the trajectory followed by the robot.

**Figure 8:**
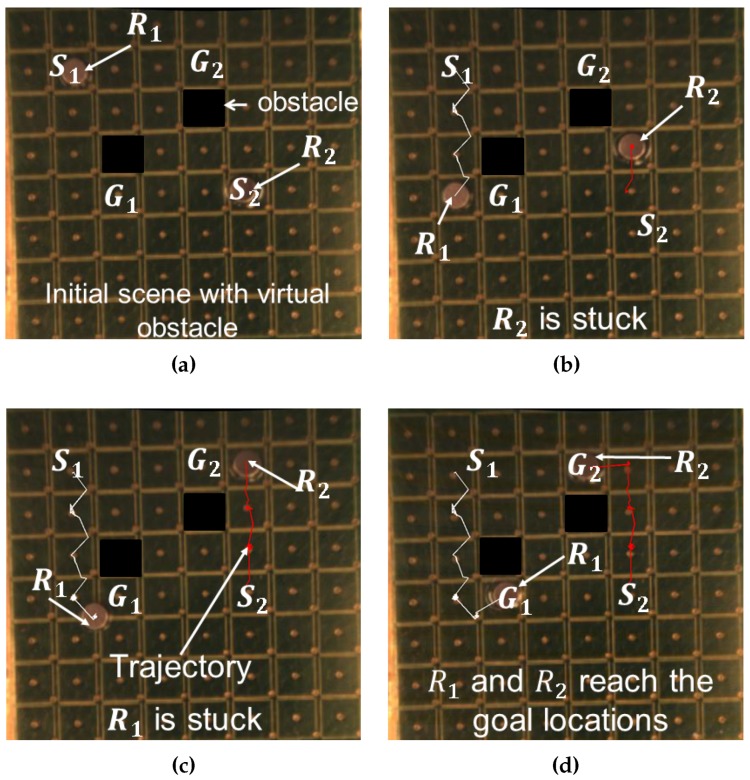
Autonomous independent navigation of two robots towards respective goal locations on an array of 64 microcoils: (**a**) Initial scene with the robots and the goal locations; (**b**) The first robot reaches a waypoint whereas the second robot is stuck due to uneven friction on the surface; (**c**)The second robot overcomes the friction to move towards the goal location whereas the first robot got stuck; (**d**) Both the robots reach their goal locations. The trajectories followed by the robots $R_{1}$ and $R_{2}$ are marked by white and red lines, respectively.

Figure 8 shows the independent navigation of two robots $R_{1}$ and $R_{2}$ towards their respective goal locations. The initial locations and goal locations are denoted by “$S_{i}$” and “$G_{i}$” (*i* = 1, 2), respectively. The friction is not uniform on the surface of the substrate. Sometimes, the robot gets stuck since the initial calculated currents are not large enough to overcome the friction (Figure 8b,c). The program can identify those scenarios if the robot gets stuck for more than usual time. In those cases, the controller increases the current in the respective coils to overcome the friction. Both the robots successfully reach their respective goal locations (Figure 8d). The white and red lines indicate the trajectories followed by robots $R_{1}$ and $R_{2}$, respectively. The trajectories are not straight lines as one can expect. The generated forces among the coils are not the same for the same input current although the design parameters are the same. This is due to the non-uniformity introduced during the PCB fabrication process. That introduces a discrepancy between the actual force and the force predicted by the model along with the measurement noise.

Although at least one of the dimensions (in this case, thickness of the robot) is less than 1 mm, the robot is not truly in the “micro” domain. Rather, we used the word “micro” to emphasis the fact that the system design and the automation approach can be extended to micro scale. With the proper modeling of the microscale forces, the control approaches can be effectively used in micro scale. We have demonstrated the automated navigation with mm-scale robots on a specialized substrate where the dimension of a coil is about 4.33 mm. However, the overall approach can be scaled down. The coil dimension can be significantly reduced to about 250 microns with the existing PCB technology. Although the reduction of coil dimension significantly reduces the resulting magnetic forces, it should be enough to drive a robot as large as 400 µm in dimension. A more sophisticated approach can be used to reduce the dimensions of the coils even more as described in [40].

We can use our planning and control approach to move µm scale robots with the 250 micron coils. However, the adhesive forces (van der Waal, capillary, *etc*.) that we have ignored for our mm-scale robots need to be modeled for effective control of micro-scale robots. Adhesive forces are difficult to estimate. However, researchers have developed experimental methods to determine these forces [41]. Better modeling of the existing external forces will improve the effectiveness of the controller.

The friction is not uniform through out the workspace due the nonuniformity of the surface introduced during the manufacturing. For the modeling, we have used constant static and dynamic friction coefficients, which may not be the case in reality. Due to this mismatch, the robot tends to get stuck in some locations as we observed in Figure 8b,c. The controller can identify these discrepancies though and gradually increases the currents to the corresponding coils to overcome these friction forces. Again, better modeling, in this case of friction, will help the controller to avoid this situation.

## 7. Conclusions

With the advent of miniaturization of high-tech products, there is a necessity for high throughput system to assemble micro and nano-scale components. Moreover, the ability to manipulate objects in high volume autonomously can revolutionize the biological experiments. Magnetic fields created by electromagnetic coils are capable of generating a wide range of forces suitable for manipulating objects in microscale. However, generating local magnetic fields and the automated actuation of multiple robots to manipulate a large number of objects independently is challenging.

In this paper, we have developed an approach for autonomous navigation of single and multiple magnetic robots in a dynamic environment. The approach starts with planning for collision free waypoints, followed by a controller to compute the required force to actuate the robots towards the waypoints, and an optimization routine to compute the required currents in the electromagnetic coils that can drive the robots. We have also presented the design of a device comprised of an array of 64 microcoils that can generate local magnetic fields for independent actuation of multiple robots. We have conducted extensive simulation and physical experiments to demonstrate the effectiveness of the approach. The parameters used in the simulation are based on extensive previous experimental characterization of the system [28].

In future, we will implement the control of orientation along with the position of the robot to navigate the robot in narrow passages in between obstacles. We will utilize the microcoil array to control multiple microrobots independently towards our ultimate goal to realize a micro-assembly manufacturing station.
